# A novel process-based model of microbial growth: self-inhibition in *Saccharomyces cerevisiae* aerobic fed-batch cultures

**DOI:** 10.1186/s12934-015-0295-4

**Published:** 2015-07-30

**Authors:** Stefano Mazzoleni, Carmine Landi, Fabrizio Cartenì, Elisabetta de Alteriis, Francesco Giannino, Lucia Paciello, Palma Parascandola

**Affiliations:** Dept. di Agraria, Università degli Studi di Napoli Federico II, Via Università 100, 80055 Portici, NA Italy; Dept. di Ingegneria Industriale, Università degli Studi di Salerno, Via Giovanni Paolo II 132, 84084 Fisciano, SA Italy; Dept. di Biologia, Università degli Studi di Napoli Federico II, Via Cinthia, 80100 Naples, Italy

**Keywords:** Yeast, System dynamics, Numerical simulations, Overflow metabolism, Autotoxicity, Metabolic shift

## Abstract

**Background:**

Microbial population dynamics in bioreactors depend on both nutrients availability and changes in the growth environment. Research is still ongoing on the optimization of bioreactor yields focusing on the increase of the maximum achievable cell density.

**Results:**

A new process-based model is proposed to describe the aerobic growth of *Saccharomyces cerevisiae* cultured on glucose as carbon and energy source. The model considers the main metabolic routes of glucose assimilation (fermentation to ethanol and respiration) and the occurrence of inhibition due to the accumulation of both ethanol and other self-produced toxic compounds in the medium. Model simulations reproduced data from classic and new experiments of yeast growth in batch and fed-batch cultures. Model and experimental results showed that the growth decline observed in prolonged fed-batch cultures had to be ascribed to self-produced inhibitory compounds other than ethanol.

**Conclusions:**

The presented results clarify the dynamics of microbial growth under different feeding conditions and highlight the relevance of the negative feedback by self-produced inhibitory compounds on the maximum cell densities achieved in a bioreactor.

## Background

Microbial cell populations growing in a closed vessel (batch culture), under suitable environmental and substrate conditions typically show an initial exponential proliferation followed by a decline in growth rate and transition to stationary phase [1]. Such dynamics, different from Malthus’ law prediction, have been essentially ascribed to either exhaustion of nutrients according to the Monod model [2], or accumulation of toxic compounds in the culture medium [3], both affecting the maximum achievable cell density under the given conditions. Accordingly, a general model of microbial population growth has to include both the effects of nutrients and the dynamics of changing environmental conditions. Such model should necessarily consider the main metabolic routes of nutrients assimilation and the possible occurrence of inhibition phenomena. These issues are addressed here on the yeast *Saccharomyces cerevisiae*, which is a reference model biological system [4] and a microorganism of major biotechnological importance [5–7].

### Metabolic shift between respiration and fermentation

Glucose catabolism of the yeast *S. cerevisiae* may follow two different pathways: aerobic respiration to CO_2_ and H_2_O or fermentation to ethanol. Furthermore, *S. cerevisiae* is a glucose-sensitive yeast [8], which means that, under aerobic conditions, it also produces ethanol when sugar concentration is high. This phenomenon has been described and explained in different ways over the years: Crabtree effect [9], or glucose effect [10] (with these two terms sometimes used as synonyms), and overflow metabolism determined by a respiratory bottleneck [11–13]. Furthermore, by analogy to a comparable phenomenon in bacteria, the term catabolite repression [14] was also used for the effect of glucose in yeast metabolism [15].

The overflow metabolism hypothesis attributes aerobic fermentation to the saturation of a limited respiratory capacity leading to an overflow reaction at pyruvate level [11]. Such effect is observed within seconds after exposure to high glucose concentrations and has been distinguished from more long-term effects concerning repression of respiration [8]. The latter has been reported to involve different signal transduction pathways activated by levels of either extracellular glucose or intracellular yet un-identified metabolites [16].

### Growth inhibition and limits to cell density

Cell densities higher than those of a typical batch culture can be obtained if the intrinsic nutrient limitation of closed systems is circumvented by performing the so-called “extended batch” or “fed-batch” culture [17]. The yeast industry has historically developed the aerated fed-batch process to produce baker’s yeast [18], while more recently, the same cultivation mode has been employed to obtain high levels of foreign proteins concentrations with recombinant strains [19].

A typical fed-batch is accomplished when the batch culture is prolonged by a continuous or intermittent supply of fresh medium to the vessel. In these cases, microbial cell density may achieve values higher than 100 g d.w. l^−1^ [20]. The theoretical maximum cell density in a microbial culture due to spatial constraints alone has been calculated to be 400 g d.w. l^−1^ [21], but considering that culture fluidity is lost when the dry cell weight is higher than 220 g d.w. l^−1^, a maximum cell density of 200 g d.w. l^−1^ has been considered reasonable [22]. It has been widely reported that maximum cell densities obtained in fed-batch cultures are generally lower than those expected, and this has been attributed to different causes: oxygen transfer limitation, accumulation of toxic by-products, increased medium viscosity and ion conductivity, and generation of CO_2_ and heat [20–23]. In other words, a microbial population cannot support an indefinite growth due to a finite environmental carrying capacity, i.e. as the population approaches the environmental limits, the growth declines and eventually stops.

In the case of *S. cerevisiae* cultured in aerated fed-batch reactors, growth rate decline has been mostly related to oxygen transfer limitations [24] especially in large bioreactors [25], while high medium viscosity, low pH and temperatures have been considered to explain the constraints observed in high-cell-density cultivations of recombinant strains [26]. However, there is also experimental evidence that yeast strains show reduced proliferative capacity despite the maintenance of optimal cultural conditions and oxygen availability [27–29].

### Mathematical models of microbial growth

Besides the experimental work, mathematical models have been proposed to describe the dynamic processes of microbial populations. The occurrence of a stationary level in microbial growth has been attributed in mathematical models to either a generic overcrowding effect [30] or explicit nutrient exhaustion as represented by Monod kinetics (for a review see [31]). Some models based on Monod’s formulation also considered the effect of either biomass concentration [32] or products [33] as growth inhibiting factors. More recently, explicit representations of yeast metabolism have been taken into account to model the aerobic fermentation process. In particular, several process-based models have been implemented based solely on the “overflow” hypothesis, i.e. a limited respiratory capacity [11, 12, 34, 35], while in other cases both overflow and repression of respiration have been taken into account [36]. Moreover, Hanegraaff et al. [37] proposed a mechanistic model of respiro-fermentative pathways associated with different responses of multiple types of glucose carriers.

A different modelling approach is that of cybernetic models [38–41] which assume that microorganisms optimize the use of available resources choosing the most convenient metabolic pathway. A recent approach is that of systems biology aiming to combine genomic, biochemical, and physiological information [42, 43]. This class of models has been specifically conceived to integrate massive amount of experimental data in order to calculate the concentration levels of all components of metabolic pathways at specific steady states [44]. However, given its level of detail this approach does not allow to represent the dynamics at population level including feedbacks from environmental conditions.

In this work, we propose a new process-based model, following the principles of System Dynamics [45] according to which a complex system can be represented by means of flows, stocks and feedback loops. The occurrence of negative feedbacks by self-produced inhibitory compounds has been demonstrated to be a basic process able to explain the onset of growth limits involved in species coexistence and pattern formation in plants [46–48]. Inspired by these modelling concepts, a new model of microbial cell growth has been developed to describe *S. cerevisiae* dynamics in a bioreactor. The model is characterised by an explicit formulation of both the metabolic shift between respiration and fermentation, represented as a function of the glycolysis process, and the self-inhibition of cell growth induced by the release of toxic by-products.

### Model description

**Fig. 1:**
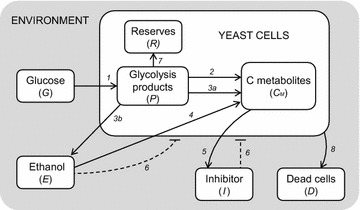
Model diagram of yeast growth. Simplified cell metabolism with explicit representation of the major metabolic pathways. *1* Glucose uptake; *2* respiration; *3a* fermentation; *3b* ethanol production by fermentation; *4* ethanol respiration; *5* secretion of inhibitory compounds; *6* inhibitory effects; *7* reserves accumulation; *8* cell death.

The model here presented describes the growth dynamics of the yeast *S. cerevisiae* cultured in a bioreactor, under aerobic conditions, with glucose as carbon and energy source. Figure 1 shows a schematic diagram of the implemented processes, providing a simplified representation of the complex network of glucose metabolism in aerobic conditions.

Glucose (*G*) is added to the growth medium according to the bioreactor feeding process. Glucose uptake is followed by the glycolytic process, which produces different products (*P*), from glucose-6-phosphate to pyruvate. These products can follow two distinct pathways for the construction of new cellular material (*C*_*M*_), either respiration or fermentation. Ethanol (*E*) produced by fermentation can also be used, instead of glucose, as carbon source for the respiratory pathway.

The main feature of the model is the inclusion of an inhibitor (*I*), representing growth-associated by-products, which accumulates in the growth medium. Ethanol and the inhibitor are assumed to separately exert a negative feedback on cell growth in a concentration-dependent way.

The second essential assumption is the key role of the glycolytic pathway in the regulation of yeast cell metabolism. High levels of glycolytic products are assumed to be responsible for (1) the activation of aerobic fermentation due to overflow metabolism, (2) repression of respiration (“glucose effect”), (3) accumulation of reserve materials (*R*), and (4) induction of mortality with accumulation of dead cells (*D*).

The setup of the glucose supply to the reactor (*Feeding*) is defined according to the different experimental settings. Metabolic processes listed in Table 1 (*Uptake, Respiration*_*P*_*, Respiration*_*E*_*, Fermentation* and *Accumulation*) are modelled based on Michaelis–Menten kinetics with respect to a given substrate and with a first order dependency on the active biomass pool (*B* = *P* + *C*_*M*_) modified according to the model assumptions made to represent *S. cerevisiae* physiology. The uptake of glucose (*Uptake*) and the consequent formation of glycolytic intermediates are also limited by product (*P*) saturation and the inhibitory effect of ethanol accumulation in the growth medium [49]. The model also allows the setting of a lag-phase term on the glucose uptake in order to account for the adaptation of yeast cells after inoculum into the bioreactor [12]. The respiration fluxes (*Respiration*_*P*_*, Respiration*_*E*_) are inhibited by the extracellular accumulation of both ethanol and the inhibitor, and also by elevated intracellular concentrations of glycolytic intermediates (glucose effect) [16]. Similarly, the fermentation flux (*Fermentation*) is inhibited by ethanol and the inhibitor, while it is only activated at elevated intracellular concentrations of glycolytic products (metabolic overflow [11]). *Accumulation* of reserve molecules (mainly glycogen) is activated at high intracellular concentrations of glycolytic intermediates (mainly glucose-6-phosphate [50]) and is also limited by product (*R*) saturation.

**Table 1:** Model processes

Equation
$$Feeding = c_{F}\cdot F_{0}\cdot { \exp }(\mu \cdot (t - t_{F} ))$$
$$Uptake = v_{G} \cdot \frac{[G]}{{k_{G} + [G]}} \cdot B\cdot\left( {1 - \frac{[P]}{{[P]_{max} }}} \right)\cdot(1 - n_{E} )\cdot lag$$
$$Respiration_{P} = v_{RP} \cdot \frac{[P]}{{k_{RP} + [P]}} \cdot B \cdot\left( {1 - n_{E} } \right)\cdot(1 - n_{I} )\cdot ge$$
$$Fermentation = v_{F}\cdot \frac{[P]}{{k_{F} + [P]}}\cdot B\cdot (1 - n_{E} )\cdot(1 - n_{I} )\cdot mo$$
$$Respiration_{E} = v_{RE}\cdot \frac{[E]}{{k_{RE} + [E]}}\cdot B \cdot (1 - n_{E} )\cdot(1 - n_{I} )\cdot ge$$
$$Accumulation = v_{A} \cdot\frac{[P]}{{k_{A} + [P]}}\cdot B\cdot\left( {1 - \frac{R}{{R_{max} }}} \right)\cdot mo$$
$$Secretion = \rho\cdot \left( {\eta_{RP} \cdot Respiration_{P} + \eta_{RE} \cdot Respiration_{E} + \eta_{FP} \cdot Fermentation} \right)$$
$$Death_{P} = d \cdot \delta \cdot P;$$ $$Death_{R} = d \cdot \delta \cdot R;$$ $$Death_{CM} = d \cdot \delta \cdot C_{M}$$

All symbols are described in Tables 2, 3 and 4.

Moreover, the production of the inhibitory compound *I* (*Secretion*) is assumed to be related to anabolic pathways hence it is expressed as a proportion of the respiration and fermentation fluxes. Cell death is assumed to be induced by elevated concentrations of glycolytic products (mainly glucose-6-phosphate [51, 52]) and is modelled as a loss (*Death*_*P*_, *Death*_*CM*_ and *Death*_*R*_) of the cellular components *P*, *C*_*M*_ and *R* respectively.

Based on the above model description, the following mass balance equations have been written:

1 $$\frac{dG}{dt} = Feeding - Uptake$$

2 $$\frac{dP}{dt} = \eta_{G} \cdot Uptake - Respiration_{P} - Fermentation - Accumulation - Death_{P}$$

3 $$\frac{dE}{dt} = \eta_{FE} \cdot Fermentation - Respiration_{E}$$

4 $$\frac{{dC_{M} }}{dt} = \eta_{RP} \cdot Respiration_{P} + \eta_{RE} \cdot Respiration_{E} + \eta_{FP} \cdot Fermentation - Secretion - Death_{M}$$

5 $$\frac{dR}{dt} = \eta_{A} \cdot Accumulation - Death_{R}$$

6 $$\frac{dI}{dt} = Secretion$$

7 $$\frac{dD}{dt} = Death_{P} + Death_{M} + Death_{R} .$$

The mathematical equations of this model are described in detail in Tables 1 and 2. The complete list of state variables and parameters along with their units and simulation values can be found in Tables 3 and 4.

**Table 2:** Symbols used in the model equations

Description	Formula
Initial feed rate	$$F_{0} = \left\{ {\begin{array}{*{20}l} {0,} & {t < t_{F} } \\ {\frac{{M_{F} \cdot \mu }}{{c_{F} \cdot y_{R} }},} & {t \ge t_{F} } \\ \end{array} } \right.$$
Glucose concentration	$\left[ G \right] = \frac{G}{V}$
Active metabolite mass	*B* = *P* + *C*_*M*_
Glycolysis products concentration	$\left[ P \right] = \frac{P}{(B + R) \cdot c}$
Ethanol negative feedback	$n_{E} = \sigma_{E}\cdot \frac{\left[ E \right]}{{\left[ E \right]_{max} }}$
Lag phase	$lag = 1 - \frac{{l_{1} }}{{1 + l_{2} \cdot { \exp }(l_{3} \cdot t)}}$
Inhibitor negative feedback	$n_{I} = \sigma_{I} \cdot\frac{[I]}{{[I]_{max} }}$
Glucose effect	$ge = \frac{1}{{1 + a \cdot { \exp }(b \cdot \left[ P \right])}}$
Metabolic overflow	$mo = 1 - ge$
Ethanol concentration	$\left[ E \right] = \frac{E}{V}$
Maximum reserves	*R*_*max*_ = *(B* + *R) r*_*max*_
Death switch	$$d = \left\{ {\begin{array}{*{20}c} {0, \left[ P \right] \le \tau } \\ {1, \left[ P \right] > \tau } \\ \end{array} } \right.$$
Medium volume in the reactor	$$V(t) = \left\{\begin{array}{ll} V_{0},& t < t_{F} \\ \frac{{M_{F} }}{{c_{F} \cdot y_{R} }}\cdot exp(\mu \cdot (t - t_{F} )), &t_{F} \le t \le t_{END}\\ \end{array} \right.$$

All other symbols are described in Tables 3 and 4.

**Table 3:** State variables initial values and simulation setup parameters

Symbol	Description	Unit	LBG H 1022^a^	CBS 8066^b^	CEN.PK113-7D^c^	CEN.PK2-1C^d^
*G*_*0*_	Glucose initial value	g	9	0	20	20
*E*_*0*_	Ethanol initial value	g	0.1	0	0	0
*P*_*0*_	Glycolysis products initial value	g	5 × 10^−5^	5 × 10^−5^	5 × 10^−5^	5 × 10^−5^
*C*_*M0*_	Carbon metabolites initial value	g	0.1	7	0.023	0.022
*I*_*0*_	Inhibitor initial value	g	0	0	0	0
*R*_*0*_	Reserve compounds initial value	g	0	0	0	0
*D*_*0*_	Dead cells initial value	g	0	0	0	0
*t*_*0*_	Time of simulation start	h	3	0	0	0
*t*_*F*_	Time of feeding start	h	–	3.2	17	15
*t*_*END*_	Time of simulation end	h	21	13	49	31; 40; 48
*c*_*F*_	Glucose concentration in feeding solution	g l^−1^	–	100	500	500
*M*_*F*_	Cell mass at beginning of feeding	g	–	3.66	3.66	4.52; 4.14; 4.14
*μ*	Feeding rate	h^−1^	–	0.3	0.16	0.1; 0.16; 0.2
*y*_*R*_	Maximum biomass yield on glucose	–	–	0.1	0.5	0.5

^a^von Meyenburg [53]; Fig. 2.

^b^Pham et al. [12]; Fig. 3.

^c^Figure 4.

^d^Figures 5, 6 and 7.

**Table 4:** Model calibrated parameters with description and simulation values

Symbol	Description	Unit	Calibration starting value	Calibrated values			
				LBG H 1022^a^	CBS 8066^b^	CEN.PK113-7D^c^	CEN.PK2-1C^d^
*v*_*G*_	Maximum uptake rate	h^−1^	3.64 [54]	3.3	5.1	5.8	5.8
*k*_*G*_	Uptake saturation constant	g l^−1^	0.18 [55]	0.2	0.2	0.27	0.27
*η*_*G*_	Uptake efficiency *P*/*G*	–	0.92 [56]	0.76	0.76	0.86	0.64
*v*_*RP*_	Maximum glycolysis products respiration rate	h^−1^	0.475 [57]	1.5	0.67	1.0	0.83
*k*_*RP*_	Glycolysis products respiration saturation constant	g l^−1^	0.033–0.035 [58]	0.24	0.21	0.24	0.18
*η*_*RP*_	Respiration efficiency *C*_*M*_/*P*	–	0.6 [56]	0.80	0.80	0.80	0.73
*v*_*F*_	Maximum fermentation rate	h^−1^	11.8 [57]	3.3	4.17	2.6	6.57
*k*_*F*_	Fermentation saturation constant	g l^−1^	0.5 [59]	0.13	0.18	0.14	0.16
*η*_*FE*_	Fermentation efficiency *E*/*P*	–	0.47 [60]	0.595	0.80	0.60	0.61
*η*_*FP*_	Fermentation efficiency *C*_*M*_/*P*	–	0.09 [61]	0.13	0.16	0.20	0.10
*v*_*RE*_	Maximum ethanol respiration rate	h^−1^	0.19 [11]	0.20	0.14	0.20	0.11
*k*_*RE*_	Ethanol respiration saturation constant	g l^−1^	0.1 [13]	0.12	0.12	0.15	0.15
*η*_*RE*_	Respiration efficiency *C*_*M*_/*E*	–	0.68 [62]	0.65	0.55	0.80	0.80
*v*_*A*_	Maximum accumulation rate	h^−1^	0.2 (Arbitrary)	0.2	0.2	0.2	0.3
*k*_*A*_	Accumulation saturation constant	g l^−1^	0.05 (Arbitrary)	0.05	0.05	0.05	0.03
*η*_*A*_	Accumulation efficiency *R*/*P*	–	0.2 (Arbitrary)	0.2	0.2	0.2	0.2
*r*_*MAX*_	Maximum reserves/cell mass ratio	–	0.25 [63]	0.3	0.3	0.3	0.3
*δ*	Death rate	h^−1^	0.017–0.032 [27]	0.05	0.05	0.05	0.1
*τ*	Death threshold	g l^−1^	0.6 (Arbitrary)	0.6	0.6	0.6	0.6
*ρ*	Secretion rate	h^−1^	0.01 (Arbitrary)	0.01	0.01	0.01	0.02
*σ*_*I*_	Sensitivity to inhibitor NF	–	1.0 (Arbitrary)	1.0	1.0	1.0	1.68
*σ*_*E*_	Sensitivity to ethanol NF	–	1.4 [49, 64, 65]	1.4	1.4	1.4	1.4
c	Cell volume/dry weight ratio	l g^−1^	0.01 [66]	0.01	0.01	0.01	0.01
*a*	Metabolic switches calibration parameter	–	2.0 × 10^−4^ (Arbitrary)	2.0 × 10^−4^	2.0 × 10^−4^	2.0 × 10^−4^	2.0 × 10^−4^
*b*	Metabolic switches calibration parameter	l g^−1^	30 (Arbitrary)	30	30	30	30
*l*_*1*_	Lag phase calibration parameter	–	0.58 (Arbitrary)	–	0.58	–	–
*l*_*2*_	Lag phase calibration parameter	–	2.0 × 10^−5^ (Arbitrary)	–	2.0 × 10^−5^	–	–
*l*_*3*_	Lag phase calibration parameter	h^−1^	5.8 (Arbitrary)	–	5.8	–	–
[*P*]_*MAX*_	Glycolysis products maximum concentration	g l^−1^	1 [67, 68]	1	1	1	1
[*E*]_*MAX*_	Ethanol maximum concentration	g l^−1^	100 [65]	100	100	100	100
[*I*]_*MAX*_	Inhibitor maximum concentration	g l^−1^	1 (Arbitrary)	1	1	1	1

^a^von Meyenburg [53]; Fig. 2.

^b^Pham et al. [12]; Fig. 3.

^c^Figure 4.

^d^Figures 5, 6 and 7.

## Results

Model simulations were compared to classic experiments of yeast growth in both batch [53] and fed-batch cultures [12], and new experiments carried out in an aerated fed-batch bioreactor with two strains belonging to the CEN.PK family [69] of the yeast *S. cerevisiae*.

### Simulation of a batch culture

**Fig. 2:**
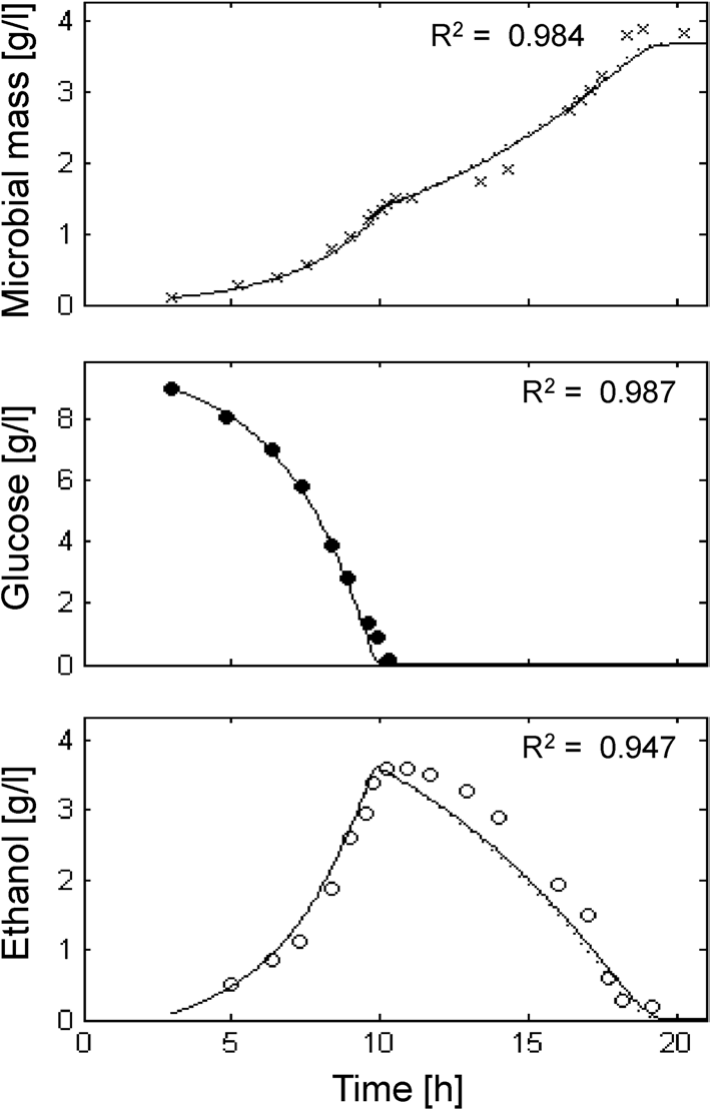
Measured vs. simulated yeast growth reproducing von Meyenburg [53] experiment. Time series of measured microbial mass (*times symbol*), glucose (*filled circle*) and ethanol (*open circle*) data vs. model simulations (*continuous lines*).

The model has been used to simulate the aerobic batch culture of *S. cerevisiae* LBG H 1022 strain growing on glucose as carbon and energy source in a classical experiment by von Meyenburg [53]. Figure 2 shows the three main variables (cell mass, glucose and ethanol concentration) monitored during the entire time course of the batch run, and the corresponding simulations. The experiment performed by von Meyenburg can be considered as a typical aerated batch culture of a glucose-sensitive yeast. In these conditions, glucose was initially fermented as shown by the accumulation of ethanol in the medium. Once glucose was exhausted (approx. after 10 h), a second exponential growth phase was observed, corresponding to the use of ethanol as substrate, according to the typical diauxic yeast growth in aerobic batch culture (Fig. 2).

The calibrated model produced a very good agreement between the experimental data and the simulated curves (Microbial mass R^2^ = 0.984; Glucose R^2^ = 0.987; Ethanol R^2^ = 0.947) describing yeast proliferation during the diauxic growth in this aerobic batch culture. In such conditions (short term culture), no evidence of inhibitory effect exerted by either ethanol or self-produced inhibitors was observed.

### Simulation of a fed-batch culture

**Fig. 3:**
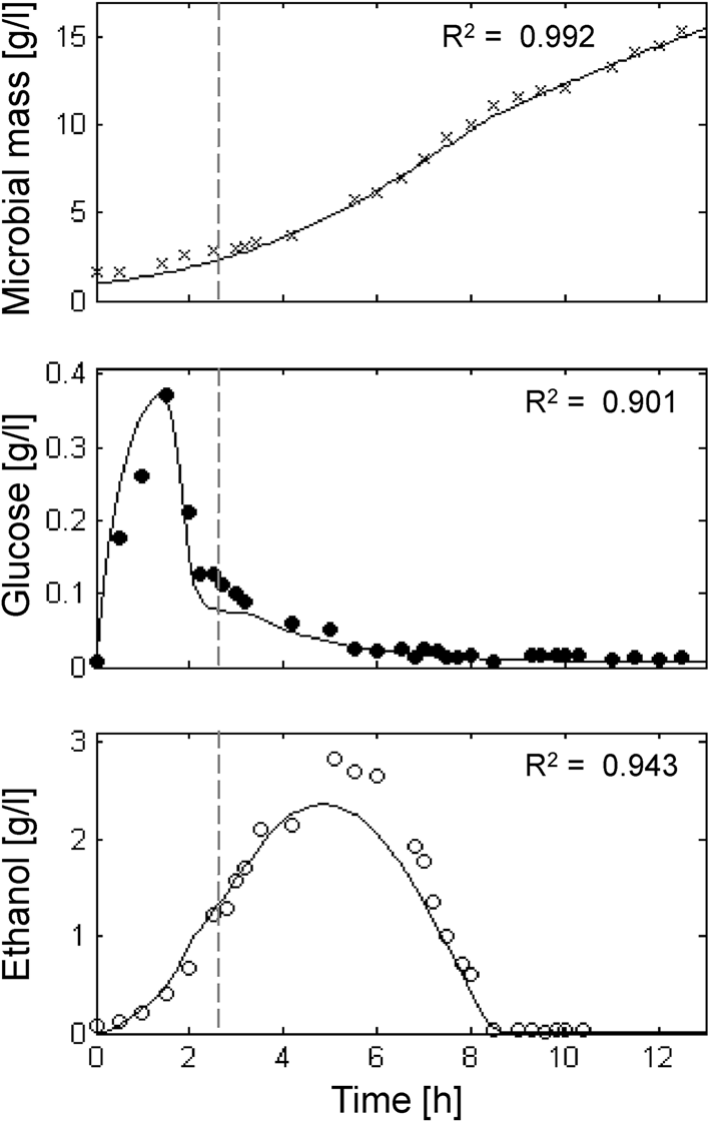
Measured vs. simulated yeast growth reproducing Pham et al. [12] experiment. Time series of measured microbial mass (*times symbol*), glucose (*filled circle*) and ethanol (*open circle*) data vs. model simulations (*continuous lines*). *Dashed vertical lines* represent the change from exponential to linear glucose feeding regime.

The aerobic fed-batch culture of *S. cerevisiae* CBS 8066, as described by Pham et al. [12], has been simulated (Fig. 3). The authors employed a two phases feeding strategy consisting of a first exponentially increasing feeding (SFR value 0.3 h^−1^, coinciding with the specific population growth rate) of 3 h, followed by a constant feeding. In the early phase of feeding (1.8 h) glucose uptake was lower than expected due to adaptation of yeast cells to the culture conditions as reported by the authors themselves. In this adaptation phase, glucose was not consumed and accumulated in the medium. Then, growth accelerated and glucose drastically decreased (Fig. 3, middle panel). Ethanol accumulation stopped due to ethanol uptake (Fig. 3, lower panel). The switch between glucose and ethanol consumption appeared without any visible diauxic lag phase in the growth profile. Finally, when ethanol was completely depleted (8 h), the growth rate slightly decreased being sustained only by the glucose feeding (Fig. 3, upper panel).

With the exception of some underestimation of the maximum level of ethanol concentration, the model was able to fit the experimental data at very significant levels (Microbial mass R^2^ = 0.992; Glucose R^2^ = 0.901; Ethanol R^2^ = 0.943). In this study case, neither experimental data nor simulation results showed evidence of growth decline related to any inhibitory effect.

### Fed-batch cultures of CEN.PK strains

**Fig. 4:**
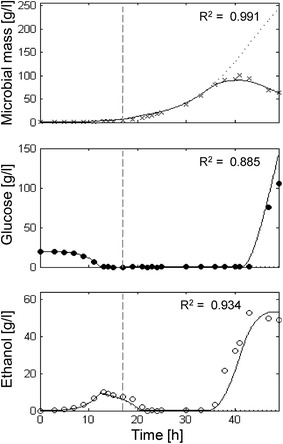
Measured vs. simulated CEN.PK prototroph strain growth in fed-batch experiment. Time series of measured microbial mass (*times symbol*), glucose (*filled circle*) and ethanol (*open circle*) data vs. model simulations (*continuous lines*). *Dashed vertical lines* represent the beginning of exponential feeding. *Dotted lines* represent simulation results without inhibitor *I* negative feedback.

**Fig. 5:**
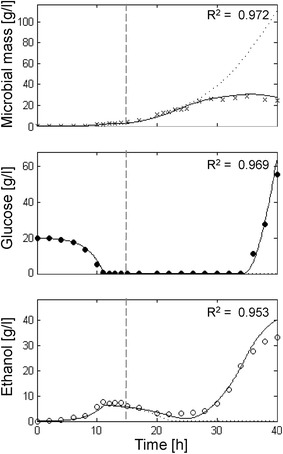
Measured vs. simulated CEN.PK auxotroph growth in fed-batch experiment. See Fig. 4 for legend. Note the different scaling of plot axes.

Experimental and simulation results of two aerobic fed-batch cultures of the *S. cerevisiae* CEN.PK113-7D and CEN.PK2-1C strains are shown in Figs. 4 and 5. Both experiments were performed starting from a batch phase followed by an exponentially increasing glucose feeding at a SFR value of 0.16 h^−1^.

In the case of the prototroph *S. cerevisiae* CEN.PK113-7D strain, the typical diauxic growth was observed during the batch phase with related glucose and ethanol dynamics (Fig. 4). Feeding started after 17 h of batch and yeast growth proceeded sustained by a predominant respiratory catabolism of both glucose and ethanol. During the early phases of feeding (up to 33 h) cell mass increased following the imposed SFR value as expected, and no residual glucose was detected in the culture medium. Then, the mass profile started to move away from the ideal one (dotted line in Fig. 4, upper panel) showing a progressive reduction of the actual growth rate if compared to the constant SFR. At the same time, ethanol began to be detected (Fig. 4, lower panel) and soon afterwards, glucose started accumulating in the culture medium (Fig. 4, middle panel). A maximum cell density of about 100 g l^−1^ was achieved after 40 h of cultivation, then cell density diminished due to the culture dilution determined by the maintenance of feeding to the reactor coupled with the reduced population growth capability. At the end of the fed-batch cultivation (50 h), a 30% of the total yeast population resulted non-viable in accordance to the simulation (data not shown).

A very good agreement between the experimental data and simulation curves was observed in this case as well (Microbial mass R^2^ = 0.991; Glucose R^2^ = 0.885; Ethanol R^2^ = 0.934). This result was achieved only if the effect of the negative feedback by the inhibitor was considered in the simulations (see difference between dotted and continuous lines in Fig. 4).

Also in the case of the auxotroph *S. cerevisiae* strain CEN.PK 2-1C (Fig. 5) the model was capable to reproduce a highly significant fitting with the experimental data (Microbial mass R^2^ = 0.981; Glucose R^2^ = 0.951; Ethanol R^2^ = 0.952). The profiles of cell mass, residual glucose and ethanol during both the batch and the feeding phases were similar to those of the prototroph (Fig. 4). However, an earlier (27 h) detachment from the ideal trend of cell mass was observed and cell density was limited to a maximum value of 30 g l^−1^ at the end of the fed-batch run (40 h). In this case, a 50% of the total yeast population resulted non-viable (data not shown).

### Model validation and application

**Fig. 6:**
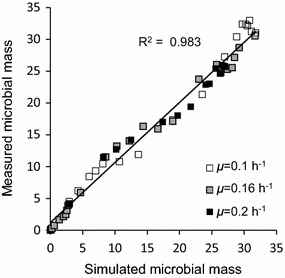
Model validation with different exponential feeding regimes. Comparison between measured vs. simulated yeast biomass data of CEN.PK 2-1C auxotroph strain from different experiments with three feeding rates (*µ*).

Further experiments with the same CEN.PK 2-1C strain were performed at different SFR values (0.1 and 0.2 h^−1^). These experiments represent a strong validation of the model since a highly significant fitting of cell mass (R^2^ = 0.983) between experimental data and simulations was maintained under all the examined feeding conditions (Fig. 6).

Moreover, an additional simulation was performed to assess feeding conditions that could avoid the switch to a fermentative metabolism during the fed-batch phase, while keeping high biomass yields. Such goal was achieved by adjusting the reactor feeding according to a logistically decreasing specific growth rate (μ*) used as SFR. This theoretical feeding profile was used to set a new fed-batch experiment with *S. cerevisiae* CEN.PK 2-1C strain.

**Fig. 7:**
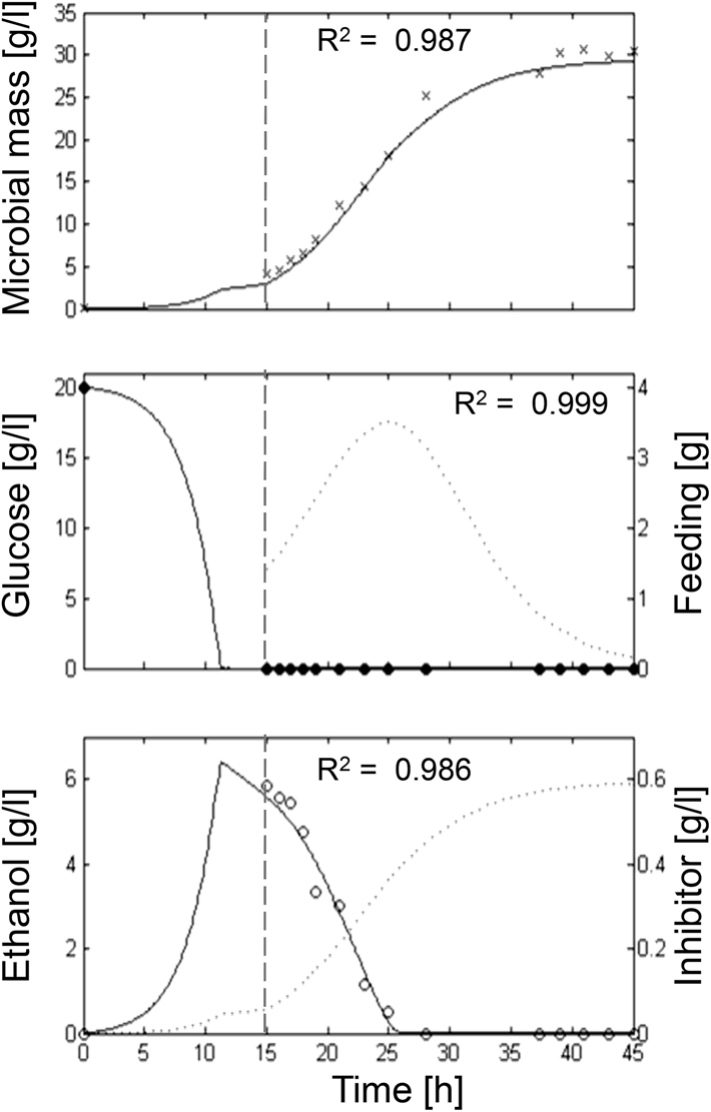
Measured vs. simulated CEN.PK auxotroph growth in fed-batch with variable feeding regime. Time series of measured microbial mass (*times symbol*), glucose (*filled circle*) and ethanol (*open circle*) data vs. model simulations (*continuous lines*). *Dashed vertical lines* represent the beginning of the feeding phase. The *dotted lines* in the *middle* and *lower panels* show the glucose feeding profile and the inhibitor simulated values respectively. Calibrated parameters for µ* were m_1_ = 0.1863, m_2_ = 0.1628 and m_3_ = 14.1092.

Resulting experimental data overlapped the simulation curves previously obtained by the model simulation for all the considered variables (biomass, glucose and ethanol) (Fig. 7).

## Discussion

The mathematical model presented has been developed to simulate yeast growth on glucose as carbon and energy source in aerated batch and fed-batch cultures, and it is based on two main assumptions. First, a central metabolic hub is assumed to regulate (1) the shift between the respiratory and fermentative pathways and (2) cell death. Second, self-inhibiting by-products and ethanol are assumed to act as concentration-dependent inhibitors of cell growth.

It is well known that pyruvate plays a central role in yeast metabolism [70], representing the starting point of the major metabolic pathways deriving from glucose uptake, hence crucial for the distribution of metabolism between respiration and fermentation [71, 72]. When glucose uptake rate increases due to its high availability, but the rate of oxidative pyruvate consumption is limited by the respiratory bottleneck [11], an overflow of pyruvate occurs leading to fermentation and ethanol production. Moreover, the “glucose repression effect” on respiration involves several signal transduction pathways activated by intracellular levels of yet un-identified metabolites deriving from glucose uptake [16]. Recent studies reported that high levels of sugars in the culture medium induce cell death accompanied with the production of reactive oxygen species (ROS) and DNA/RNA degradation in both animals [73, 74] and yeasts [51]. Furthermore, Granot and Dai [52] demonstrated that glucose and fructose phosphorylation (the first steps of glycolysis) in yeast cells is essential to activate sugar induced cell death.

Taking into account the abovementioned findings, the model attributes the role of central metabolic hub to pyruvate and/or other intermediate metabolites produced along the glycolytic pathway (collectively represented by the variable *P*), which at high concentrations progressively trigger the activation of the fermentative pathway (“overflow metabolism”), the repression of respiration (“glucose effect”) and finally cell death.

Usually, fermentation is described as less efficient than respiration because it produces less ATP per mole of glucose consumed [75] rising the “apparent paradox” [71] of cells switching to the less efficient aerobic fermentation in the case of overflow metabolism. According to our model, it can be speculated that overflow metabolism may represent an escape strategy to avoid accumulation of intracellular sugars up to toxic levels, with fermentation ensuring the necessary lowering of intracellular sugars concentration.

The *P*-driven metabolic shift implemented in the model was perfectly capable to describe yeast behaviour both in batch and fed-batch cultures. In the batch culture reported in Fig. 2, the initial glucose concentration in the medium was high and fermentation was the only possible metabolism at the beginning of cultivation. Then, when the glucose level dropped, cells resumed the respiratory metabolism and a second phase of growth was observed at the expense of ethanol, showing the typical diauxic growth. In short, fed-batch cultures (Fig. 3) after the initial fermentative phase induced by the excess of glucose in the medium, the cell population constantly increased growing at a $\mu$ value very close to the specific feeding rate (SFR), without any production of ethanol or glucose accumulation in the medium. Differently, in extended fed-batch cultures (Figs. 4, 5), despite the optimal conditions of both aeration and nutrition in the bioreactor, after several hours of feeding (about 18 and 12 h for CEN.PK 113-7D and CEN.PK2-1C strains, respectively) the growth rate started to decline. In these experiments, the sequential accumulation of ethanol and glucose occurred after the start of growth decline. This implies that the complete metabolic shift to fermentation was a consequence rather than the cause of the observed reduced growth performance. The model tackles this phenomenon by representing the secretion of a growth-linked inhibitory compound, which accumulates in the culture medium thus affecting the growth rate. It is worth noticing that, in the performed experiments, the given SFR was lower than the critical value determined for the CEN.PK strains [27], which should have produced a specific growth rate $\mu$ equal to the imposed SFR [76] and neither glucose nor ethanol should have accumulated in the medium. Simulations confirmed that population growth would continue according to the imposed SFR in the case of absence of such inhibitory compound (dotted lines in Figs. 4, 5). It is also interesting to notice that in the validation experiments (Fig. 6) the maximum cell density is achieved at the lowest SFR (0.1) and decreases at progressively higher feeding rates (0.16 and 0.2). The explanation for this behaviour could be related to an anticipated metabolic shift towards fermentation in the latter cases with consequent lower efficiency.

Historically, the concept of self-poisoning affecting the end-point of growth dates back to some experimental observations on bacterial cultures made by Rahn [77]. Then, the idea of self-poisoning/autotoxicity regulating cell proliferation has been disregarded, since nutrient depletion was recognized as the prevailing phenomenon in common laboratory batch cultures, overcoming the possible concomitant effect of accumulation of inhibitors [3]. Therefore, growth of microbial cell cultures has been almost exclusively related to nutrient depletion by the classic Monod equation [2] and models derived [78].

Our results show that the new proposed model is capable of simulating the typical diauxic growth of different strains of *S. cerevisiae* in a batch culture. The simulations of the classical von Meyenburg’s experiment (Fig. 2) and the batch phases of the experiments with CEN.PK strains (Figs. 4, 5) confirm that, in a closed vessel, the stationary phase of a yeast population has to be ascribed only to the depletion of the limiting substrate (first glucose and then ethanol). Indeed, in a batch culture the accumulation of inhibitors (including ethanol) apparently do not achieve critical concentration values (Fig. 7, lower panel). On the contrary, nutrient exhaustion cannot explain the growth decline observed in prolonged fed-batch cultures. In these cases, the model clearly demonstrates that the decline cannot be related to the concentration of ethanol, but rather to a negative feedback exerted by the accumulation of other inhibitory compounds in the culture medium. The last experiment performed with variable feeding profile clearly shows the occurrence of the growth decline without the production of ethanol, being related only to the accumulation of the inhibitor (Fig. 7, lower panel). It has to be noted that the accumulation of the inhibitor perfectly resembles the population biomass growth since its secretion is calculated as a fixed proportion of the yeast growth (see equations in Table 1). As shown in Figs. 4 and 5, during the initial phase of exponential growth, cell mass increases following the glucose feeding curve. Meanwhile, the inhibitory compounds accumulate in the culture medium until they start to slow down the respiration flux, which is the active metabolic pathway in this phase of the aerobic fed-batch culture. As respiration decreases, while glucose uptake and glycolysis are still substantial, internal *P* concentration increases and activates fermentation with consequent ethanol production. However, also the fermenting cells are affected by the inhibitory compounds, with a continuous decline in growth rate and consequent progressive accumulation of glucose in the medium. The growth-inhibited cells, exposed to the exponentially increasing glucose fed to the bioreactor, face a further increase of the internal *P* concentration, which finally induced cell death. Indeed, at the end of the two fed-batch cultures of CEN.PK strains 30% and 50% of the prototrophic and auxotrophic populations respectively resulted non-proliferating, in agreement with simulation results (data not shown). On the other hand, it is worth noting that in the experiment characterized by a variable feeding, the cells were never exposed to high glucose concentrations (Fig. 7), and consequently their internal *P* concentration never reached levels sufficient to induce ethanol production and then cell death. This is consistent with the common empirical approach followed in some fed-batch cultures, where the occurring limitations are faced by keeping a constant feeding after the initial exponential phase [12, 24]. Interestingly, the lower cell density achieved by the auxotroph strain compared to the prototroph was simulated by the model also by the increase of the negative feedback effect due to a presumable higher secretion of the self-inhibitors in the culture medium. We can speculate that this could be related with the observation of the enhanced permeability of auxotrophic strains [28, 79].

The presence of inhibitory toxic compounds in microbial cell cultures has been widely reported, for instance unrestricted aerobic fed-batch cultivations of bacteria such as *Escherichia coli* and *Bacillus subtilis* accumulate acetate and propionate, respectively [20]. In the case of *S. cerevisiae*, acetate is also produced during glucose fermentation [75] and it has been reported to be toxic [80, 81]. However, acetate appeared together with ethanol in the experiments presented and it was found only in small amounts after the onset of growth rate decline [28].

Ethanol, the main end-product of glucose fermentation, exerts its toxic effect at multiple levels on yeast cells, acting as a non-competitive inhibitor of growth at concentrations higher than 5% v/v. [49, 64]. Both model and experimental results presented in this work excluded that ethanol accumulation could be responsible for the initial growth decline observed during prolonged fed-batch cultures, whereas the inhibition due to high ethanol concentration contributed to limit the cell density finally achieved along with the other modelled inhibitory compounds. In addition, it is worth noting that the exhausted medium collected at the end of the fed-batch cultures, once ethanol was removed by evaporation under vacuum, was still inhibitory for *S. cerevisiae* growth (data not shown).

## Conclusions

The model presented was able to reproduce the dynamic behaviour of several yeast strains growing both in batch and fed-batch cultures. It is interesting that a very simplified System Dynamics model has been sufficient to capture the major dynamics of yeast metabolism and proliferation under different feeding conditions. From the applied point of view, the good prediction performance of the model suggests its possible use for the optimization of feeding strategies aimed to maximize biomass yield and glucose saving. From a theoretical point of view, these results support the importance of negative feedback processes in the understanding of microbial growth processes. Further investigation will be necessary to determine the chemical nature of the inhibitory compounds, other than ethanol and acetate, involved in such negative feedback, and the related mechanisms of action. The identification of the inhibitory compounds will be performed through GC- or LC–MS and NMR methodologies and the study of the mechanisms of action will start with the analysis of available “omics” data, especially those concerning gene expression of *S. cerevisiae* during diauxic shift and stationary phases [82]. Moreover, it will be interesting to verify the model behaviour in other experimental conditions with different carbon and energy sources, investigating the dynamics of reserves accumulation, induction of quiescence and cell death.

## Methods

### Numerical simulations

The model has been first developed in the SIMILE (Simulistics Ltd) visual modelling environment in order to facilitate the discussion within the multidisciplinary team during the implementation phases. Then, the mathematical equations were integrated using MATLAB R2012b (the MathWorks) with a variable order solver (ode15s) based on the numerical differentiation formulas (NDFs) particularly efficient with stiff problems [83].

The model calibration was performed by minimizing the sum of the squared errors (SSE)

$$SSE = \sum\nolimits_{i = 1}^{{n_{1} }} {\left( {C_{Mi} - C_{Mi}^{*} } \right)^{2} } + \sum\nolimits_{i = 1}^{{n_{2} }} {\left( {G_{i} - G_{i}^{*} } \right)^{2} } + \sum\nolimits_{i = 1}^{{n_{3} }} {\left( {E_{i} - E_{i}^{*} } \right)^{2} } ,$$

where n_1_, n_2_, n_3_ are the number of samples per observed outputs, $C_{Mi}$, $G_{i}$, $E_{i}$, are the values of the *i*th measured outputs and $C_{Mi}^{*}$, $G_{i}^{*}$, $E_{i}^{*}$, are the values of the *i*th outputs predicted by the model. The minimization was performed by using the fminsearch MATLAB routine which implements a Nelder–Mead simplex algorithm [84]. Units and initial values of the state variables and the parameters used to setup the simulated experiments are reported in Table 3. Units and values of the calibrated parameters for each simulated yeast strain are reported in Table 4.

Data regarding four *S. cerevisiae* strains, obtained from literature and the new experimental work have been used to test the model. In detail, model simulations were compared to classic experiments of growth in batch [53] and fed-batch culture [12] and to the results from fed-batch cultures of two yeast strains, a prototroph and an auxotroph. These belong to the CEN.PK family of *S. cerevisiae*, which is largely used in industry and academic research [69, 85]. The reported experiments were performed in an aerated bioreactor fed at different specific feeding rates (SFR), namely 0.1, 0.16 and 0.2 h^−1^.

The calibration procedure was performed on four of the available datasets (von Meyenburg’s batch [53], Pham’s fed-batch [12], fed-batch cultures at SFR = 0.16 h^−1^ of the two CEN.PK strains). The resulting parameter values are reported in Table 4. Validation tests were performed comparing model simulations with the experimental data of the auxotroph CEN.PK strain grown at both lower (0.1 h^−1^) and higher (0.2 h^−1^) SFRs. In order to test the model predictive capability a further validation experiment was performed. Logistically decreasing specific growth rate (µ*) was assumed as follows:

$$\mu^{*} = m_{1} - \frac{{m_{1} }}{{1 + { \exp }( - m_{2}\cdot (t - t_{F} - m_{3} ))}}$$

where m_1_, m_2_ and m_3_ are calibration parameters. An optimization procedure was performed in MATLAB to find the values of m_1_, m_2_ and m_3_ to maximise the biomass yield and minimise the ethanol production. Specifically, we used the MATLAB fminsearch routine to minimize the following objective function (OF):

$$OF = \frac{1}{{{ \hbox{max} }(C_{M} (t))}} + \mathop \sum \limits_{i = 0}^{t} E_{i}$$

where *t* is the number of simulation time steps and *max*(*C*_*M*_(*t*)) is the maximum value of *C*_*M*_ achieved during the simulation. The resulting values of µ* where used as SFR to calculate the reactor feeding as reported in the next section. The other parameters were kept constant for all validation tests and are reported in Tables 3 and 4, last column.

The following expression:

$$\left[ M \right] = \frac{{P + C_{M} + R + D}}{V}$$

was used to represent the microbial mass concentration as derived from optical density readings including both viable biomass (*P* + *C*_*M*_ + *R*) and dead cells (*D*) in the culture medium volume (*V*).

### Fed-batch cultures of the CEN.PK strains

The strains used for the experimental work were: the prototroph *S. cerevisiae* CEN.PK 113-7D (*MATa URA3 HIS3, LEU2 TRP1 MAL2*-*8c SUC2*) and the auxotroph *S. cerevisiae* CEN.PK2-1C (*MATa ura3*-*52 his3*-*D1 leu2*-*3,112 trp1*-*289 MAL2*-*8c SUC2*). They were purchased at EUROSCARF collection (www.uni-frankfurt.de/fb15/mikro/euroscarf).

Fed-batch cultures of the CEN.PK strains were performed in a 2.0 l working volume of a stirred fermenter, Bioflo110 (New Brunswick Scientific). The vessel initially contained 1 l of a medium prepared according to Verduyn et al. [86], with 2% w/v glucose, vitamins and trace elements, and supplemented with 10 g l^−1^ of casamino acids (BD Bacto TM Casamino Acids, BectonDickinson & Co., Sparks, MD 21152 USA). In the case of the auxotroph strain the medium was supplemented with uracil, histidine, leucin, triptophan according to [87] so to fully cover yeast request for the entire fermentation run.

The reactor was inoculated with an adequate aliquot of a pre-culture still growing in the exponential phase and prepared in the same medium above described, to give an initial O.D._590_ of 0.04. After an initial batch phase, where glucose was depleted, the reactor was fed with a solution containing 50% w/v glucose and salts, trace elements, glutamic acid and vitamins according to [29]. The runs have been carried out at three different specific feed rates (SFR), namely 0.10, 0.16, 0.20 h^−1^, by supplying the reactor with an exponentially increasing feeding, according to:

$$F\left( t \right) = F_{0}\cdot { \exp }({SFR \cdot t})$$

where *F*(*t*) is the time-dependent feed rate (m^3^ h^−1^), *F*_*0*_ is the initial feed rate and SFR is the specific feed rate (h^−1^) which, in ideal conditions [76], is equal to the population specific growth rate (*µ*).

The three different SFR values were all below the *µ* critical value in correspondence of which ethanol begins to be produced [69].

To test the predictive capability of the model another fed-batch culture of the *S. cerevisiae* CEN.PK2-1C strain was performed. The run was carried in the same cultural conditions as described above, but logistically decreasing the specific growth rate (µ*), as reported in the paragraph “Model description”. The resulting values of µ* where used as SFR to calculate the reactor feeding *F*(*t*).

In all the fed-batch cultures, oxygen was supplied to the reactor by air sparging. The dissolved oxygen tension (DOT) was kept at 30% air saturation by the cascade system, by controlling the impeller speed, or, when this reached its maximum value (1,000 rpm) by enrichment with pure oxygen. The culture pH was maintained at 5.00 by automatic addition of 2N KOH during batch phase, and 10% v/v NH_4_OH during fed-batch phase. The foam level in the fermenter was controlled by the automatic addition of antifoam B (Sigma Aldrich) solution at 10% v/v.

Cell mass was determined by optical density at 590 nm (O.D._590_) and dry weight determination. The calibration curve relating O.D._590_ values to biomass density provided a correlation factor of 2.30 O.D._590_ per g l^−1^. Cell viability during fed-batch runs was determined by viable count (in triplicate) on YPD (yeast extract 1%, bactopeptone 2%, dextrose 2% w/v) agar plates incubated at 30°C for 48 h.

Samples were hourly withdrawn from cultures, filtered on 0.45 μm GF/A filters (Millipore, Bedford, MA, USA) and the filtrates analysed to determine residual glucose and ethanol concentrations in the culture medium (g l^−1^). Glucose and ethanol were determined by enzymatic d-glucose assay (GOPOD Format) and Ethanol-enzymatic kit from Megazyme (Megazyme International, Ireland Ltd.), respectively. All the samples were analysed in triplicate showing a standard deviation always lower than 5%.

## References

[1] Buchanan R (1918). Life phases in a bacterial culture. J Infect Dis.

[2] Monod J (1949). The growth of bacterial cultures. Annu Rev Microbiol.

[3] Bailey JE, Ollis F (1986). Biochemical engineering fundamentals.

[4] Botstein D, Fink GR (2011). Yeast: an experimental organism for 21st Century biology. Genetics.

[5] Nevoigt E (2008). Progress in metabolic engineering of *Saccharomyces cerevisiae*. Microbiol Mol Biol Rev.

[6] Porro D, Gasser B, Fossati T, Maurer M, Branduardi P, Sauer M (2011). Production of recombinant proteins and metabolites in yeasts: when are these systems better than bacterial production systems?. Appl Microbiol Biotechnol.

[7] Hong KK, Nielsen J (2012). Metabolic engineering of *Saccharomyces cerevisiae*: a key cell factory platform for future biorefineries. Cell Mol Life Sci.

[8] Fiechter A, Seghezzi W (1992). Regulation of glucose metabolism in growing yeast cells. J Biotechnol.

[9] De Deken RH (1966). The Crabtree effect: a regulatory system in yeast. J Gen Microbiol.

[10] Holzer H (1976). Catabolite inactivation in yeast. Trends Biochem Sci.

[11] Sonnleitner B, Käppeli O (1986). Growth of *Saccharomyces cerevisiae* is controlled by its limited respiratory capacity: formulation and verification of a hypothesis. Biotechnol Bioeng.

[12] Pham HT, Larsson G, Enfors SO (1998). Growth and energy metabolism in aerobic fed-batch cultures of *Saccharomyces cerevisiae*: simulation and model verification. Biotechnol Bioeng.

[13] Sonnleitner B, Hahnemann U (1994). Dynamics of the respiratory bottleneck of *Saccharomyces cerevisiae*. J Biotechnol.

[14] Magasanik B (1961). Catabolite repression. Cold Spring Harb Symp Quant Biol.

[15] Gancedo JM (1998). Yeast carbon catabolite repression. Microbiol Mol Biol Rev.

[16] Westergaard SL, Oliveira AP, Bro C, Olsson L, Nielsen J (2007). A systems biology approach to study glucose repression in the yeast *Saccharomyces cerevisiae*. Biotechnol Bioeng.

[17] Pirt SJ (1975). Principles of microbe and cell cultivation.

[18] Reed G, Peppler H, Reed G, Peppler H (1973). Baker’s yeast production. Yeast technology.

[19] Porro D, Sauer M, Branduardi P, Mattanovich D (2005). Recombinant protein production in yeasts. Mol Biotechnol.

[20] Riesenberg D, Guthke R (1999). High-cell-density cultivation of microorganisms. Appl Microbiol Biotechnol.

[21] Riesenberg D (1991). High-cell-density cultivation of *Escherichia coli*. Curr Opin Biotechnol.

[22] Lee SY (1996). High cell-density culture of *Escherichia coli*. Trends Biotechnol.

[23] Shiloach J, Fass R (2005). Growing *E. coli* to high cell density—a historical perspective on method development. Biotechnol Adv.

[24] Van Hoek P, De Hulster E, Van Dijken JP, Pronk JT (2000). Fermentative capacity in high-cell-density fed-batch cultures of baker’s yeast. Biotechnol Bioeng.

[25] Fu Z, Verderame TD, Leighton JM, Sampey BP, Appelbaum ER, Patel PS (2014). Exometabolome analysis reveals hypoxia at the up-scaling of a *Saccharomyces cerevisiae* high-cell density fed-batch biopharmaceutical process. Microb Cell Fact.

[26] Mattanovich D, Gasser B, Hohenblum H, Sauer M (2004). Stress in recombinant protein producing yeasts. J Biotechnol.

[27] Landi C, Paciello L, de Alteriis E, Brambilla L, Parascandola P (2011). Effect of auxotrophies on yeast performance in aerated fed-batch reactor. Biochem Biophys Res Commun.

[28] Landi C, Paciello L, de Alteriis E, Brambilla L, Parascandola P (2015). High cell density culture with *S. cerevisiae* CEN.PK113-5D for IL-1β production: optimization, modeling, and physiological aspects. Bioprocess Biosyst Eng.

[29] Paciello L, de Alteriis E, Mazzoni C, Palermo V, Zueco J, Parascandola P (2009). Performance of the auxotrophic *Saccharomyces cerevisiae* BY4741 as host for the production of IL-1beta in aerated fed-batch reactor: role of ACA supplementation, strain viability, and maintenance energy. Microb Cell Fact.

[30] Verhulst PF (1838). Notice sur la loi que la population suit dans son accroissement. Corresp Math Phys.

[31] Jannasch HW, Egli T (1993). Microbial growth kinetics: a historical perspective. Antonie Van Leeuwenhoek.

[32] Contois DE (1959). Kinetics of bacterial growth: relationship between population density and specific growth rate of continuous cultures. J Gen Microbiol.

[33] Ierusalimsky ND, Neronova NM (1965). Quantitative relationship between metabolic products concentration and growth rate of microrganisms. Ann USSR Acad Sci.

[34] Barford JP, Hall RJ (1981). A mathematical model for the aerobic growth of *Saccharomyces cerevisiae* with a saturated respiratory capacity. Biotechnol Bioeng.

[35] Coppella SJ, Dhurjati P (1990). A mathematical description of recombinant yeast. Biotechnol Bioeng.

[36] Lei F, Rotbøll M, Jørgensen SB (2001). A biochemically structured model for *Saccharomyces cerevisiae*. J Biotechnol.

[37] Hanegraaf PPF, Stouthamer AH, Kooijman SALM (2000). A mathematical model for yeast respiro-fermentative physiology. Yeast.

[38] Ramkrishna D, Blanch HW, Papoutsakis ET, Stephanopoulos G (1983). Foundations of biochemical engineering. Foundations of biochemical engineering, (ACS Symposium Series).

[39] Jones KD, Kompala DS (1999). Cybernetic model of the growth dynamics of *Saccharomyces cerevisiae* in batch and continuous cultures. J Biotechnol.

[40] Giuseppin ML, van Riel NA (2000). Metabolic modeling of *Saccharomyces cerevisiae* using the optimal control of homeostasis: a cybernetic model definition. Metab Eng.

[41] Di Serio M, Aramo P, de Alteriis E, Tesser R, Santacesaria E (2003). Quantitative analysis of the key factors affecting yeast growth. Ind Eng Chem Res.

[42] Förster J, Famili I, Fu P, Palsson BØ, Nielsen J (2003). Genome-scale reconstruction of the *Saccharomyces cerevisiae* metabolic network. Genome Res.

[43] Castrillo JI, Zeef LA, Hoyle DC, Zhang N, Hayes A, Gardner DCJ (2007). Growth control of the eukaryote cell: a systems biology study in yeast. J Biol.

[44] Adiamah DA, Handl J, Schwartz J-M (2010). Streamlining the construction of large-scale dynamic models using generic kinetic equations. Bioinformatics.

[45] Forrester JW (1961). Industrial dynamics.

[46] Bonanomi G, Giannino F, Mazzoleni S (2005). Negative plant-soil feedback and species coexistence. Oikos.

[47] Mazzoleni S, Bonanomi G, Giannino F, Incerti G, Dekker SC, Rietkerk M (2010). Modelling the effects of litter decomposition on tree diversity patterns. Ecol Model.

[48] Carteni F, Marasco A, Bonanomi G, Mazzoleni S, Rietkerk M, Giannino F (2012). Negative plant soil feedback explaining ring formation in clonal plants. J Theor Biol.

[49] Stanley D, Bandara A, Fraser S, Chambers PJ, Stanley GA (2010). The ethanol stress response and ethanol tolerance of *Saccharomyces cerevisiae*. J Appl Microbiol.

[50] Wilson WA, Roach PJ, Montero M, Baroja-Fernández E, Muñoz FJ, Eydallin G (2010). Regulation of glycogen metabolism in yeast and bacteria. FEMS Microbiol Rev.

[51] Granot D, Levine A, Dorhefetz E (2003). Sugar-induced apoptosis in yeast cells. FEMS Yeast Res.

[52] Granot D, Dai N (1997). Sugar induced cell death in yeast is dependent on the rate of sugar phosphorylation as determined by *Arabidopsis thaliana* hexokinase. Cell Death Differ.

[53] Von Meyenburg HK (1969). Energetics of the budding cycle of *Saccharomyces cerevisiae* during glucose limited aerobic growth. Arch Mikrobiol.

[54] Heyland J, Fu J, Blank LM (2009). Correlation between TCA cycle flux and glucose uptake rate during respiro-fermentative growth of *Saccharomyces cerevisiae*. Microbiology.

[55] Weusthuis RA, Pronk JT, van den Broek PJ, van Dijken JP (1994). Chemostat cultivation as a tool for studies on sugar transport in yeasts. Microbiol Rev.

[56] Christen S, Sauer U (2011). Intracellular characterization of aerobic glucose metabolism in seven yeast species by 13C flux analysis and metabolomics. FEMS Yeast Res.

[57] Van Urk H, Schipper D, Breedveld GJ, Mak PR, Scheffers WA, van Dijken JP (1989). Localization and kinetics of pyruvate-metabolizing enzymes in relation to aerobic alcoholic fermentation in *Saccharomyces cerevisiae* CBS 8066 and Candida utilis CBS 621. Biochim Biophys Acta.

[58] Kresze GB, Ronft H (1981). Pyruvate dehydrogenase complex from baker’s yeast. 1. Purification and some kinetic and regulatory properties. Eur J Biochem.

[59] Postma E, Verduyn C, Scheffers WA, Van Dijken JP (1989). Enzymic analysis of the crabtree effect in glucose-limited chemostat cultures of *Saccharomyces cerevisiae*. Appl Environ Microbiol.

[60] Paciello L, Zueco J, Landi C (2014). On the fermentative behavior of auxotrophic strains of *Saccharomyces cerevisiae*. Electron J Biotechnol.

[61] Van Maris AJA, Geertman JMA, Vermeulen A, Groothuizen MK, Winkler AA, Piper MDW (2004). Directed evolution of pyruvate decarboxylase-negative *Saccharomyces cerevisiae*, yielding a C2-Independent, glucose-tolerant, and pyruvate-hyperproducing yeast. Appl Environ Microbiol.

[62] Paalme T, Elken R, Vilu R, Korhola M (1997). Growth efficiency of *Saccharomyces cerevisiae* on glucose/ethanol media with a smooth change in the dilution rate (A-stat). Enzyme Microb Technol.

[63] Polakis ES, Bartley W (1966). Changes in dry weight, protein, deoxyribonucleic acid, ribonucleic acid and reserve and structural carbohydrate during the aerobic growth cycle of yeast. Biochem J.

[64] Yang K-M, Lee N-R, Woo J-M, Choi W, Zimmermann M, Blank LM (2012). Ethanol reduces mitochondrial membrane integrity and thereby impacts carbon metabolism of *Saccharomyces cerevisiae*. FEMS Yeast Res.

[65] Walker GM, Walker GM (1998). Yeast growth. Yeast physiology and biotechnology.

[66] Haddad SA, Lindegren CC (1953). A method for determining the weight of an individual yeast cell. Appl Microbiol.

[67] Yang YT, Bennett GN, San KY (2001). The effects of feed and intracellular pyruvate levels on the redistribution of metabolic fluxes in *Escherichia coli*. Metab Eng.

[68] Diderich JA, Raamsdonk LM, Kruckeberg AL, Berden JA, Van Dam K (2001). Physiological properties of *Saccharomyces cerevisiae* from which hexokinase II has been deleted. Appl Environ Microbiol.

[69] Van Dijken J, Bauer J, Brambilla L, Duboc P, Francois J, Gancedo C (2000). An interlaboratory comparison of physiological and genetic properties of four *Saccharomyces cerevisiae* strains. Enzyme Microb Technol.

[70] Pronk J, Steensma H, Van Dijken J (1996). Pyruvate metabolism in *Saccharomyces cerevisiae*. Yeast.

[71] Alberghina L, Mavelli G, Drovandi G, Palumbo P, Pessina S, Tripodi F (2012). Cell growth and cell cycle in *Saccharomyces cerevisiae*: basic regulatory design and protein-protein interaction network. Biotechnol Adv.

[72] Youk H, van Oudenaarden A (2009). Growth landscape formed by perception and import of glucose in yeast. Nature.

[73] Yu T, Sheu S-S, Robotham JL, Yoon Y (2008). Mitochondrial fission mediates high glucose-induced cell death through elevated production of reactive oxygen species. Cardiovasc Res.

[74] MacFarlane M, Robinson G, Cain K (2012). Glucose—a sweet way to die. Cell Cycle.

[75] Gancedo C, Serrano R, Rose AH, Harrison JS (1989). Energy-yielding metabolism. The yeasts.

[76] Enfors SO, Häggström L (1998). Bioprocess technology: fundamentals and applications.

[77] Rahn O (1932). Physiology of bacteria.

[78] Panikov NS (1995). Microbial growth kinetics.

[79] Paciello L, Landi C, Zueco J, Parascandola P (2013). Production in fed-batch reactor of *Bacillus subtilis* LipaseA immobilized on its own producer *Saccharomyces cerevisiae* cells. Chem Eng Trans.

[80] Casatta N, Porro A, Orlandi I, Brambilla L, Vai M (2013). Acetate but not propionate induces oxidative stress in bakers’ yeast *Saccharomyces cerevisiae*. Biochim Biophys Acta.

[81] Semchyshyn HM, Abrat OB, Miedzobrodzki J, Inoue Y, Lushchak VI (2011). Acetate but not propionate induces oxidative stress in bakers’ yeast *Saccharomyces cerevisiae*. Redox Rep.

[82] Galdieri L, Mehrotra S, Yu S, Vancura A (2010). Transcriptional regulation in yeast during diauxic shift and stationary phase. Omics J Integr Biol.

[83] Shampine LF, Reichelt MW (1997). The MATLAB ODE suite. SIAM J Sci Comput.

[84] Lagarias JC, Reeds JA, Wright MH, Wright PE (1998). Convergence properties of the Nelder–Mead simplex method in low dimensions. SIAM J Optim.

[85] Nijkamp JF, van den Broek M, Datema E, de Kok S, Bosman L, Luttik MA (2012). De novo sequencing, assembly and analysis of the genome of the laboratory strain Saccharomyces cerevisiae CEN.PK113-7D, a model for modern industrial biotechnology. Microb Cell Fact.

[86] Verduyn C, Postma E, Scheffers W, Van Dijken J (1992). Effect of benzoic acid on metabolic fluxes in yeasts: a continuous-culture study on the regulation of respiration and alcoholic fermentation. Yeast.

[87] Pronk JT (2002). Auxotrophic yeast strains in fundamental and applied research. Appl Environ Microbiol.

